# Supplementary material for: Removing auto-activators from yeast-two-hybrid assays by conditional negative selection
Source: Sci Rep. 2021 Mar 9;11:5477. doi: 10.1038/s41598-021-84608-9 (PMC7943551; doi:10.1038/s41598-021-84608-9)
Supplement: Supplementary file 3 — Supplementary Information 3. [file 41598_2021_84608_MOESM3_ESM.docx]

**Supplementary Material**

**Title:** Removing auto-activators from yeast-two-hybrid assays by conditional negative selection

**Authors:** Devendra Shivhare^1^, Irene Julca^1^, Pawel Gluza^2,3^, Marek Mutwil^1,2*^


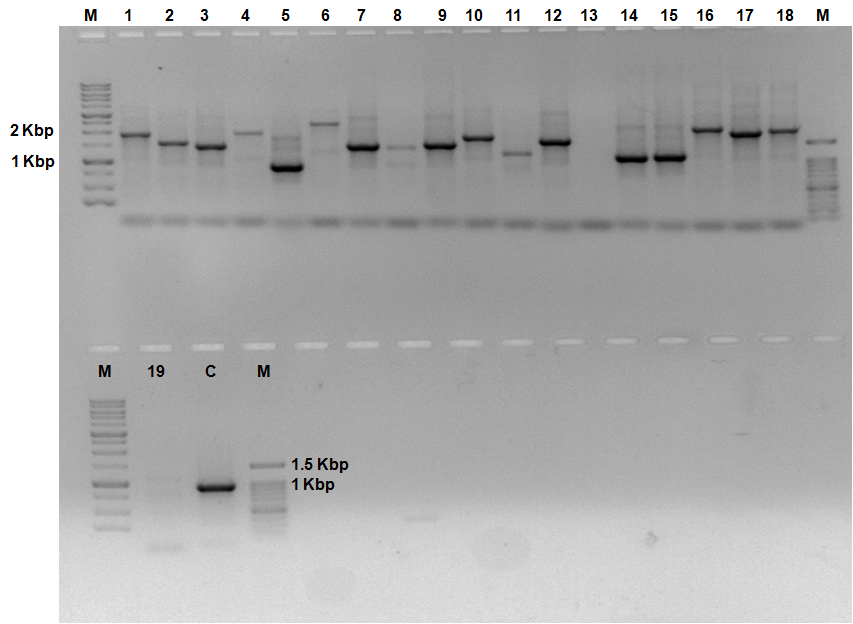


**Figure S1. PCR analysis of 19 colonies cloned into the entry vector.** The quality of the library was assessed by picking a total of 19 colonies for colony PCR run with the M13F+R primers. The PCR reaction was loaded onto 1% agarose. M: marker, 1-19: colonies, C: pDONR vector control.


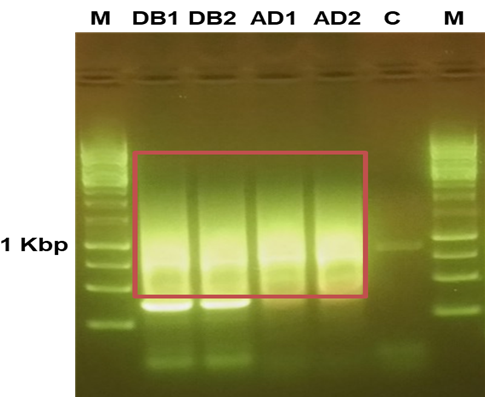


**Figure S2.** The plasmids were isolated from BD and AD libraries and PCR-amplified. The smear that was used to purify DNA from the gel and sent for NGS analysis is indicated by the red box.
